# Understanding recovery in the context of lived experience of personality disorders: a collaborative, qualitative research study

**DOI:** 10.1186/s12888-015-0572-0

**Published:** 2015-07-31

**Authors:** Steve Gillard, Kati Turner, Marion Neffgen

**Affiliations:** 1 Population Health Research Institute, St George’s, University of London, Cranmer Terrace, London, SW17 0RE UK; 2 South West London & St George’s Mental Health NHS Trust, Springfield University Hospital, Glenburnie Way, London, SW17 7DJ UK

**Keywords:** Recovery, Personality disorders, Lived experience, Discourse, Qualitative research, Collaborative research, Co-production of knowledge

## Abstract

**Background:**

Concepts of recovery increasingly inform the development and delivery of mental health services internationally. In the UK recent policy advocates the application of recovery concepts to the treatment of personality disorders. However diagnosis and understanding of personality disorders remains contested, challenging any assumption that mainstream recovery thinking can be directly translated into personality disorders services.

**Methods:**

In a qualitative interview-based study understandings of recovery were explored in extended, in-depth interviews with six people purposively sampled from a specialist personality disorders’ service in the UK. An interpretive, collaborative approach to research was adopted in which university-, clinical- and service user (consumer) researchers were jointly involved in carrying out interviews and analysing interview data.

**Results:**

Findings suggested that recovery cannot be conceptualised separately from an understanding of the lived experience of personality disorders. This experience was characterised by a complexity of ambiguous, interrelating and conflicting feelings, thoughts and actions as individuals tried to cope with tensions between internally and externally experienced worlds. Our analysis was suggestive of a process of recovering or, for some, discovering a sense of self that can safely coexist in both worlds.

**Conclusions:**

We conclude that key facilitators of recovery – positive personal relationships and wider social interaction – are also where the core vulnerabilities of individuals with lived experience of personaility disorders can lie. There is a role for personality disorders services in providing a safe space in which to develop positive relationships. Through discursive practice within the research team understandings of recovery were co-produced that responded to the lived experience of personality disorders and were of applied relevance to practitioners.

**Electronic supplementary material:**

The online version of this article (doi:10.1186/s12888-015-0572-0) contains supplementary material, which is available to authorized users.

## Background

### Recovery and mental health

Recovery-orientated approaches to mental health care have their origins in the mental health service consumer and survivor movements (for example Wellness Recovery Action Planning, [1]). These approaches offer an alternative to clinical models of recovery traditionally focused on cure from disease and reduction in symptoms, suggesting instead that recovery concerns a broader picture of living well with mental health issues [2]. A recent review of recovery literature identified five inter-locking processes that can be said to characterise recovery: empowerment and reclaiming control over one’s life; rebuilding positive personal and social identities; connectedness (both personal and wider aspects of social inclusion); hope and optimism about the future; finding meaning and purpose in life [3]. From the 1990s recovery has been advocated as a set of principles upon which mental health services should be organised [4]. From the turn of the millennium UK health policy began to suggest that mental health service providers adopt a recovery approach to the delivery of services [5]. Ten years on the UK mental health strategy [6] stipulated recovery as an outcome against which the performance of provider organisations will be measured, defining recovery as people’s ‘greater ability to manage their own lives, stronger social relationships, a greater sense of purpose, the skills they need for living and working, improved chances in education, better employment rates, and a suitable and stable place to live’.

### Recovery and personality disorders

In the UK concepts of recovery applied in mainstream mental health services have begun to be translated into approaches to delivering services for personality disorders. A ‘capabilities framework’ for the treatment of personality disorders [7] indicates that staff need to develop skills to support people experiencing personality disorders with their recovery. Similarly, National Institute for Health and Clinical Excellence (NICE) guidance on the treatment of Borderline Personality Disorder [8] makes reference to the possibility of recovery. Neither of these documents specifies how recovery is to be understood in the context of personality disorders. Understandings of recovery informing current service developments in mental health are grounded in an established qualitative, narrative literature exploring mental illness and recovery [9, 10]. Not only have experiential recovery accounts been absent from the personality disorders’ literature; there is also a paucity of qualitative research that describes personality disorders from the experiential perspective [11].

### Recovery from what, to what? Personality disorders; a contested discourse

The appropriateness of ‘mainstream’ understandings of recovery in the context of lived experience of personality disorders has been challenged,’self-discovery’ being proposed as one alternative understanding [12]. Recovery has been described as a highly individualised concept responding to the different needs and aspirations of each individual [13]. There is a tension inherent in the application of existing understandings of recovery to the personality disorders context in the absence of a well-developed phenomenological literature articulating individual lived experiences of personality disorders. Indeed understandings of personality disorders are in themselves contested. Historically personality disorders have been viewed by some health professionals and the general public as ailments of character – of personality – as opposed to ‘true’ mental illnesses, and as such, untreatable [14]. Contrastingly, a growing body of evidence now exists that demonstrates that medical-type recovery (symptom alleviation) from personality disorders can be observed over time [15], and that long-term treatment benefits can be achieved where appropriate treatments are developed [16, 17]. These more recent developments have not always acknowledged voices which have long maintained that treatment outcomes for personality disorders are good, most notably heard within the Therapeutic Community movement [18] where treatments have been pioneered since the 1950s. There is some conceptual equivalence in existing understandings of mental health recovery and Therapeutic Community approaches; for example, ‘social connectedness’ [3] overlaps to some extent with the idea of ‘community as doctor’ [19]. However it is important to note that recovery concepts currently being applied across mainstream mental health services have emerged from neither the theory and practice of personality disorders treatment, nor the lived experience of personality disorders. There is a need for detailed qualitative research, at an individual experiential level, that addresses the question of whether and how concepts of recovery can be meaningfully applied in the context of personality disorders.

### Aims

This article will report on a qualitative research project which aimed to explore understandings of recovery from the perspectives of people with lived experience of personality disorders. Since we undertook this study the findings of two other qualitative research projects that explored understandings of recovery in the context of personality disorders have been published [20, 21]. We consider those studies in the discussion below, and note differences in methodological approach and in the resulting findings that suggest that our paper usefully complements this emerging literature.

## Methods

### Study design

This was an exploratory, qualitative interview-based study that sought to investigate understandings of recovery of people with a lived experience of personality disorders. We adopted a collaborative approach to research; this was because we set out to produce findings that would be relevant both to people with lived experience of personality disorders and to clinical teams working in and developing personality disorders services. As such elicitation and interpretation of data needed to respond to experiential and clinical perspectives. The research team (the authors) comprised a university based researcher with expertise in supporting collaborative research (SG), a service user (consumer) researcher (KT) – a researcher with lived experience of personality disorders and of accessing a range of personality disorders services who subsequently trained as a researcher – and a clinical researcher (trainee psychiatrist) with experience of, and a commitment to working in personality disorders services (MN). This explicitly interpretative approach – in acknowledging the perspective of the researchers in the ‘making sense’ process (see, for example, [22]) – was in contrast to the more phenomenological approaches employed by Katsakou and colleagues [20], and Castillo, Ramon and Morant [21]. Those studies comprised 42 individual interviews, and 14 group and 20 individual interviews respectively, and produced knowledge that can be reasonably claimed to generalise to the populations studied. We were interested in how the frames of reference of the different researchers on our team mediated our interpretations of the lived experience of our participants, and so enriched our situated understandings [23] of recovery and personality disorders from academic, service user and clinical perspectives. To achieve this we undertook a small number of extended, in-depth one to one interviews and adopted a reflexive, dialogic approach to the analysis process [24]. Ethical approval for the study was given by the Wandsworth (UK) National Health Services Research Ethics Committee. Written informed consent was obtained from all study participants.

### Sample

We conducted interviews with six people. As noted above, as an exploratory, inductive study we were interested in a smaller number of in-depth, data rich cases from which to generate possible understandings of recovery and the lived experience of personality disorders. Given that we were not seeking in this study to make knowledge claims that generalised to specified populations we did not attempt to achieve saturation of our data by recruiting to a large sample. The reliability or wider validity of our findings would need to be tested in a subsequent study designed for that purpose.

Inclusion criteria for the study were i) adults of working age, and ii) current use of a specialist personality disorders service in a London mental health Trust (governmental health service provider). The specialist service provided open access, self-referral peer support groups for people living in the community who either had a diagnosis of personality disorder(s) or who self-identified with the behaviours, thoughts and emotions associated with personality disorders.

It is important to note here that all personality disorders research takes place in the wider context of ongoing debates about the relevance and usefulness of personality disorders diagnoses as tools to manage access to appropriate healthcare services [11, 25]. In an effort to improve access, a number of specialist personality disorders services in the UK do not require formal diagnoses. As is also the case with the work of Castillo, Ramon and Morant [21], our findings therefore relate to a population that meets service access criteria defined by generic difficulties associated with having a diagnosis of personality disorder [26], in contrast to a diagnostically defined population [20].

We selected our sample purposively to ensure variation in the sample and so elicit a range of possible understandings of recovery [27]. The service user researcher visited the peer support groups and talked to staff and service users about the research; interviews were arranged with service users who consented to participate in the study. Our sample comprised three men and three women, 3 of whom were aged 26–35, 1 aged 36–45, 1 aged 46–55 and 1 aged 56–65. Five participants self-identified as White while one female interviewee identified her ethnicity as Other (non-specified). Three participants self-reported as having a diagnosis of Borderline Personality Disorder, and one a diagnosis of Avoidant Personality Disorder. One participant said that they had been given a personality disorder diagnosis but did not specify which diagnosis. One participant had no personality diagnosis but self-reported as experiencing personality disorder; this participant reported a diagnosis of Body Dismorphia Disorder. Interviewees attended three different peer support groups in different localities, and half of them had also used other specialist personality disorders services. All of the interviewees had been attending the peer support groups for two or more years and all had also received various forms of non-specialised mental health care over a long period (e.g., community mental health services, psychiatric inpatient care). It is relevant to add that the Trust we recruited from had a high profile recovery policy that reflected national policy in the UK, with all staff receiving mandatory recovery training and all service users having access to personal recovery plans. As such it is likely that our sample had at least some familiarity with concepts of recovery as typically articulated in UK mental health services.

### Data collection

With permission of the authors, we used an interview schedule developed for a project that explored how the concept of recovery was understood by people using a range of different specialist mental health services [28]; see Additional file 1. The schedule was designed to explore individuals’ personal understandings and experiences of recovery, and how the services they used had supported their recovery. The schedule had been developed by a team comprising both clinical and service user researchers and was designed to be used by a clinical and a service user researcher interviewing together. Researchers alternated in asking sets of questions. Both researchers asked additional follow-up questions where they felt something the interviewee had said might be explored in more depth. The approach was designed to ensure that data collected were not shaped predominantly by the priorities of either clinical or service user researcher. Interviews were between one and a half, and two and a half hours in length. One interviewee was interviewed twice, at their request, for a total of two and a half hours. Interviews were digitally recorded and transcribed verbatim by the interviewers.

### Data analysis

Research has shown that service user researchers can have an impact on the analysis of qualitative interview data [29]. To ensure that interpretations of all members of the research team informed our analysis we devised a thematic analysis process in a number of stages with members of the team sharing the tasks. In particular we wanted to ensure that insights into personality disorders from the perspectives of both the clinical and service user researcher informed key decisions in the analysis. The role of the university researcher was to provide a more distanced perspective on the data and to help manage the analysis process. Team meetings were held throughout to monitor and guide progress. The process is illustrated in Table 1 below:

**Table 1:** The analysis process

Stage in the analysis process	Team involvement
1. Preliminary coding of a sample section of different parts of different interviews	All researchers
2. Meeting to generate a set of themes to analyse complete interviews	All researchers
3. Thematic analysis of the complete set of interviews	Clinical and service user researchers
4. Producing interpretive documents for each theme from individual researchers’ perspectives	Clinical and service user researchers
5. Producing joint interpretive documents for each theme	All researchers
6. Writing up research findings; analytical commentary and quotes from the interviews	All researchers

We used complementary thematic and framework analysis techniques [30]. Stages one and three used conventional coding techniques [31] to generate, and code data to categories. Stages two and five used framework approaches to chart, explore meaning in, and refine emerging themes as a team [32]. Stages three – collating data to themes – and four – producing analytical narrative around the data – were undertaken by clinical and service user researchers in order to prioritise those interpretive perspectives in the analysis process. The university researcher rejoined the process in stage five in order to help integrate those perspectives. Stage six was characterised by iterative rounds of writing of interpretative narrative around the data as an active part of the research process [33]. As such stages four to six of the process are explicitly interpretive, rather than simply descriptive of the data [31]. It is the interpretive narrative we develop in stage six that we present below, illustrated with reported data from the interviews.

## Results

Through the process described above we produced a set of fifteen descriptive themes organised into three sections. The iterative process of writing and rewriting within the team – stage 6 process – resulted in a set of interpretive themes that cut across our emerging understanding of recovery. The final set of themes is illustrated in Table 2 below:

**Table 2:** Thematic organisation of the data

Section	Initial themes	Final themes
1. The lived experience of personality disorders	1.1 The internal world	The internal world
	1.2 The external world	The external world
	1.3 Diagnosis	Diagnosis
2. Personality disorders and recovery	2.1 Personal understandings of recovery	Recovering or discovering the self – reconciling the internal and external worlds
	2.2 Acceptance	Recovery and discovery – doing things differently
	2.3 Positive feelings about recovery	Recovery and discovery – feeling and thinking differently
	2.4 Relationships and recovery	
	2.5 Recovery and society	
	2.6 Obstacles to recovery	
	2.7 Goals	
3. Treatment and support	3.1 Medication	
	3.2 Specialist	
	3.3 Mainstream	
	3.4 Therapy	
	3.5 Support	

In order to answer our questions about their understandings of recovery, all participants spoke in depth about their lived experience of personality disorders. It was clear that we would not be able to explore the concept of recovery without first articulating that lived experience of personality disorders. Given the lack of existing literature describing that experience, noted above, it seemed important to report that data here. We report detailed findings on supporting recovery in personality disorders services elsewhere [34], focussing in this paper on our aim of understanding recovery in the context of lived experience of personality disorders.

### The lived experience of personality disorders

**Fig. 1:**
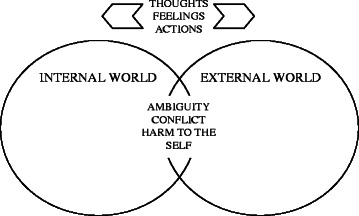
The lived experience of personality disorders. Outward facing arrows indicate the polarising thoughts, feelings and actions reported by participants with respect to internally and externally experienced worlds. The intersection of the circles represents experiences of ambiguity, conflict and harm to the self

In articulating their lived experiences of personality disorders, interviewees seemed to us to be describing a continuous tension within the self between the experiences of an internal and an external world; a complexity of ambiguous, interrelating and conflicting feelings, thoughts and actions as the individual tries to live in and cope with both worlds. Interviewees reported feeling alienated by a hostile outside world and needing to isolate themselves within their internal world in order to feel safe, often at the cost of a punitive or harmful relationship with the self. This articulation of the lived experience of personality disorders is represented diagrammatically in Fig. 1 below and explored in the analysis that follows:

#### The internal world

Interviewees told us they often experienced feelings of extreme isolation and detachment:

*… very isolated, extremely isolated … I couldn’t trust my own judgement, or anything like that … I get very detached. It’s like I’m sort of up here looking down and ‘what’s happened?’* (Int 5)

Interviewees recognised that this sometimes self-imposed isolation could be harmful and could lead to further feelings of alienation:

*… way I see it is I isolate myself when I feel I have to, I’ll go mad if I don’t isolate myself and then when I isolate myself too long I feel like I am going mad anyway so I can’t work out how I can survive properly …* (Int 1)

Interviewees spoke about intense and polarised emotions, of rapid changes in mood, and of the unpredictability and chaos that this brought to their lives:

… *it can change instantly how I’m feeling … I could be fine one minute and the next minute I would just go down, straight down … you’re walking along sometimes, a trapdoor opens and you just fall through the trapdoor and you can’t get back up again …* (Int 3)

Interviewees spoke about feeling vulnerable, and of the panic and anxiety they experienced in their everyday lives as a result:

*Well, I’m a very sort of scared person, very anxious, I panic at the slightest things sometimes …* (Int 6)

Interviewees frequently referred to feeling unsure of their own sense of self; of whether or not they were ‘normal’:

*I find I have a very uncertain sense of self, so it’s hard to know, to have hope in yourself when you don’t have a clear idea of who you are …* (Int 4)

*I think that to the day that I drop dead, whenever that is, I will still be, I don’t know what normal is …* (Int 1)

Interviewees also talked about not knowing what was wrong with them and of not knowing how to get on in the outside world as a result:

*I didn’t know what was going on. I just knew it wasn’t right because my behaviour wasn’t right … I spent a lot of time wondering what was wrong with me, thinking why can’t I be happy or why can’t I go and get a job … everything was why and I had no answers to the whys …* (Int 2)

Interviewees described a negative and punitive relationship with the self, characterised by feelings of self-hatred, low self-esteem and self-criticism:

*… feelings of complete inadequacy and self-loathing, self-hate and generally nothing positive … very self-critical, just blame myself for everything, just feel that I let everybody down and … it all snowballs, doesn’t it? You know, ‘I’m useless, I’m worthless’ …* (Int 3)

Often these self-critical feelings led to self-punishment, or self-destructive or high risk behaviours:

*I punish myself and I hurt* … *my last serious attempt was last summer and I took 250 olanzapine and I stockpiled a whole lot of medication … was in intensive care for about five or six days …* (Int 4)

*I tend not to, when things are getting difficult, go down to the pub because … I know where that ends … I end up in a bit of a mess or … somewhere trying to score drugs …* (Int 5)

#### The external world

Interviewees described how they perceived the external world as an unpredictable, and potentially harmful and hostile place:

*I’m not happy where I live and I haven’t been for a long time and I have disturbance from the neighbours and very paranoid about it … (Int 3)*

*… feeling that I’ll be destroyed. I feel as though I live in a culture that is making me ill …* (Int 1)

This perception seemed to us to be influenced by interviewees’ internal worlds; external experience and internal state of mind seemed to relate in a vicious circle:

*My perception of people involved in my care undergoes like a paradigm shift. The people who I once thought were caring and supportive become … tyrants who are trying to manipulate me and erode my freedom … human beings are all evil and corrupt and everyone’s out to take advantage of me …* (Int 4)

Most interviewees told us that they felt the only way of feeling safe was to isolate themselves from the external world:

*When I get more paranoid, I isolate myself, I don’t go out for weeks and months on end. I just barricade myself indoors, stay indoors …* (Int 2)

However that decision to withdraw was often a dilemma, interviewees recognising that they were isolating themselves but feeling that they had little choice:

*I know that anything that I don’t invite into my life will have a negative impact on my mind so I choose not to do that and people can sort of sit there and go ‘oh, well you’re isolating yourself’. Well no, I’m actually protecting myself from a whole load of crap that’s going to start going round in my head …* (Int 5)

Interviewees described becoming practised at limiting their contact with the outside world as a coping strategy:

*I only feel that I’m feeling better because … I don’t have to engage with the world … I can go outside but then I can come home if I feel I can’t cope. That sounds sort of very pathetic but actually it feels like the only way I can control my feelings …* (Int 1)

Interviewees also pointed out that actual experience of stigma was behind their decisions to withdraw:

*… if you imagine floating through life and not really being able to connect with anyone at all because you have to hide yourself, you have to put on a mask to show the world, that you’re not mentally ill because it, there’s such a stigma behind it* (Int 5)

Interviewees told us about difficulties in relationships, and often conceived of how they interacted with others as a barrier to engagement with the outside world:

*I think expectations … what other people might expect from you, like if you go out … instead of sitting in a corner you’ve got to participate in playing a game of darts or a game of pool but I’m quite happy in my corner with my drink of coke … you don’t live up to their expectations then, that’s probably why I haven’t got any friends …* (Int 6)

Interviewees spoke of a sense of failing in the outside world as a result of the difficulties and struggles they experienced with fitting in:

*At the moment, someone asks what have you done with your life, you know, I have done absolutely nothing, I left school at twelve, drink, drugs, prison, come out, a locked ward, that’s it. I can’t say that to somebody that’s been to university, has got a good job … I said once in an AA meeting about being in prison and someone went ‘argh, that’s disgusting’, and that fucking kicked me in the teeth. (Int 2)*

#### Diagnosis

We collected extensive data on interviewees’ experiences of, and attitudes towards diagnosis. We report here examples that both reinforce the lived experience of personality disorders we have illustrated above, and conversely enable the processes of recovery described in the following section. Interviewees suggested that receiving a diagnosis of personality disorder could increase their isolation from the wider world because of the stigma from friends, family and medical professionals that resulted:

*… my GP, her attitude changed completely when I got this diagnosis and she was much more guarded and wary of me … when I finally disclosed that I was BPD … my friends stopped returning my calls …* (Int 4)

Ideas about untreatability present in the literature, which potentially impact on expectations for recovery, were also reflected in interviewee accounts:

*… having a diagnosis of borderline personality disorder I’m constantly told by people that I’m not going to get any better … my doctor said: ‘you won’t get better after this’ …* (Int 5)

There was ambivalence towards receiving a diagnosis, with interviewees also reporting that the knowledge and information they received as a result helped them to understand aspects of their experience:

*… before I found out my diagnosis I was floating around thinking ‘what’s wrong with me … This isn’t just depression?’ … as soon as I found out … I started reading up on it and it got me to a point where I can understand myself … helped me to accept myself, which then increases self-esteem, self-value …* (Int 5)

### Understandings of recovery in the context of lived experience of personality disorders

**Fig. 2:**
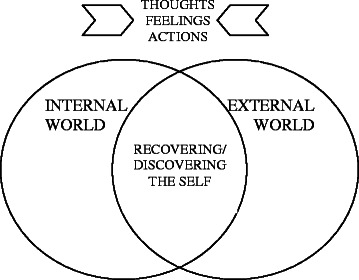
Understandings of recovery in the context of lived experience of personality disorders. Inward facing arrows indicate the thoughts, feelings and actions reported by participants that suggest the potential to reconcile internally and externally experienced worlds. The intersection of the circles represents processes of recovering or discovering a sense of self that coexists in both internally and externally experienced worlds

We asked interviewees what recovery meant to them, and they answered very much in the context of the lived experience of personality disorders described above. Interviewees talked about recovery in terms of thinking, feeling and acting in different ways that suggested to us the potential for internal and external worlds to become, to a certain extent, reconciled. We suggest that this represents a process of recovering or, for some, discovering a sense of self where they could safely coexist in both worlds, without damage to the self or of having to retreat once again into the internal world. This understanding of recovery is represented in Fig. 2 below and described in the analysis that follows:

#### Recovering or discovering the self – reconciling the internal and external worlds

Interviewees talked about both a readiness to change – of being unable to carry on as before – and a sense of recognition or acceptance of ‘the way they were’ as necessary precursors to recovery:

*I found that I was doing the same thing over and over again … unless you understand yourself I don’t think that … you can recover. Really it’s been a question of … being able to face myself and look at myself warts and all … ‘what am I going to do about it?’ … I’ve recognised that if I carried on thinking like that I’m just gonna keep tripping myself up and I’m never gonna have anything nice to say about myself*. (Int 5)

The limitations of leading an isolated lifestyle were also acknowledged:

*But I think spending too much time on my own, not going out, could be quite dangerous for me …* (Int 2)

Interviewees did talk about recovery in terms of developing the skills and strategies necessary to exercise control over their lives, reflecting aspects of ‘mainstream’ mental health recovery. However we focus here on reporting the distinctiveness of what interviewees told us about their understandings of recovery in the context of lived experience of personality disorders. Much of this specific understanding of recovery was about finding ways of being – of thinking, feeling and acting – that enabled them to live in the outside world:

*For me personally it means … sort of reintegration into the community and sort of mainstream society … to combat the feeling of alienation that I experience, maybe diminishing the frequency of self‐destructive behaviour, suicide attempts … hopefully stopping the self‐harming altogether … fewer inpatient admissions … establishing personal relationships, re-entering education and eventually finding paid employment I suppose would be the ultimate objective …* (Int 4)

*I’ve met over the past couple of years, seen people actually, not say recovering fully but actually gaining more confidence through contact with other people and actually getting out and doing things …* (Int 3)

At the same time, interviewees recognised the problems that increased interaction with wider society could present them with:

*… too many negative things … start to make me feel a bit unstable, so even though I’m trying to be stronger and I feel stronger maybe it wouldn’t take that much to destabilise me …* (Int 1)

Interviewees described the conscious choices that they had to make about how much interaction with the outside world they could cope with:

*… it will require a certain amount of me hiding again but I don’t have to hide from everyone … in order for me to be able to get back into normal society I’m going to have to step out of that and feel quite uncomfortable for a period of time while I adjust to it …* (Int 5)

Interviewees acknowledged that the process of re-engaging, through work and wider interaction, would have to be a gradual process in order to cope with setbacks and to develop confidence:

*I’m trying … to do some part time work … I couldn’t go into a full-time job straightaway. It will have to be a little step at a time … I will need to build up my confidence. I’ve got a little bit but it will need to improve …* (Int 6)

#### Recovery and discovery – doing things differently

Interviewees described future goals and aspirations focused on improved social interaction on a number of levels:

*I’d like to re‐enter education and finish my degree ‘cos I haven’t quite finished that yet … maybe establish more interpersonal relationships, real friendships with people, and maybe a romantic relationship would be nice …* (Int 4)

*… if one could get that together and go out … just once or twice a week, go to the local pub …* (Int 6)

Interviewees talked about a range of health and wellbeing changes to their lifestyle, and in particular the importance of physical activity as a means of engaging bodily with the wider world:

*Healthy diet and exercise, socialising, having some sort of structure to your day, whether it’s working an hour every two weeks or volunteering or, there’s lots I’d want to do …* (Int 3)

*… just to get out and get on my bike … you can still be part of society and the world without having to communicate with people, so I find cycling really useful …* (Int 2)

Recognising and avoiding situations associated with negative and destructive actions was very important to some interviewees:

*… taking drink out of it and things like that and keeping myself out of potentially stressful situations sort of limits the impact of it …* (Int 5)

All interviewees recognised the importance of enduring and trusting relationships, and identified positive changes they had made in terms of making and keeping friends:

*I would put that down to the quality of friendships that I’ve made. I haven’t made tons of friends or anything like that but I’ve made some really absolute diamond friendships … the thing that’s really made a difference is being able to socialise …* (Int 5)

One interviewee in particular spoke about the importance of having friendships with people who would not judge them:

*I think people need friendship, they need somebody that they feel they can turn to that won’t necessarily turn away from them in disgust or anything or judge them …* (Int 1)

However interviewees also recognised that setbacks in recovery could result from feeling judged, rejected or not understood in their relationships:

*I think if you’re put in a situation where people would get to know me, I think that could have a negative effect on my recovery and not a positive one.* (Int 2)

#### Recovery and discovery – feeling and thinking differently

In parallel, interviewees spoke of a range of internal changes – to their feelings, thoughts and sense of self – which they would like to achieve. These included being able to control and manage feelings and thoughts, and becoming more self-confident, self-assured and positive:

*I’d like not to feel the way I do most of the time, there’s always an underlying feeling of doom and gloom which I’ve just carried with me for years and years. I’d like that to not be as pronounced, not be as damaging to me, which it is, so, to change feelings definitely. There’s a lot of confusion in my head, and I just get very confused, I find I can’t organise thoughts and in turn I can’t organise my life …* (Int 3)

Some interviewees spoke about successful processes of changing the ways they thought and felt:

*Usually it’s only a few minutes because I have to kick in my coping mechanisms and talk to myself and say ‘this is part of your illness, it’ll pass in a minute’, what thoughts are there that shouldn’t be there or that aren’t doing me any good so I have to, like, sit down, identify it, face it and then take steps to counteract it …* (Int 5)

Other interviewees referred to a continuing sense of negativity, either undermining progress they had made towards recovery or inhibiting them from addressing issues of isolation:

*… there’s no positivity there, no sort of sense of even wanting to recover, it’s just … can’t be bothered, what’s the point … I have a lot of fear involved that it’s not going to work that I’m just not going to change, there is always that great fear …* (Int 3)

Interviewees spoke about the need to balance the extreme and polarised feelings they experienced in order to maintain their recovery:

*The only thing that changes is the fluctuation of my emotions so I have to bear that in mind as well and not get full of despair … so I have to sort of cushion it between getting too high and getting too low. I have to try and keep this sort of balance going which is really, really hard work …* (Int 5)

Many interviewees talked about how the disconnection they experienced from their feelings inhibited their ability to make changes:

*… you don’t feel no achievement … they do encourage you, praise you but, with the way our minds are working we don’t feel like we’ve done nothing …* (Int 6)

Interviewees described the constant struggle to make progress as exhausting and potentially defeating:

*I’m not weak but … some of these feelings are … so deeply ingrained in me that however much I try, and I am trying, I’m not sure that I can turn everything round because your history’s your history, however much you try …* (Int 1)

However interviewees also described what we understood to be a virtuous cycle in their recovery, where forming positive relationships could reinforce positive changes in their thoughts and feelings, and vice versa:

*… a more sort of constant and solid idea of self and personal identity, it’s not something you can do in isolation … by having good, positive relationships where you get feedback and they reinforce the sort of positive aspects of yourself then you’re able to become more functional, more normal and integrate more easily into society …* (Int 4)

*I spent so many years not giving a damn about anybody … If I upset someone it would be like, that’s your stuff not mine. I couldn’t care less. Today I do think about other people and take other people’s feelings into account … it makes me feel good when I help others …* (Int 2)

## Discussion

### The lived experience of personality disorders

In this paper we found it necessary to understand first our interviewees’ lived experiences of personality disorders as the context of our enquiry; to explore understandings of recovery from the perspectives of people with lived experience of personality disorders. Our resulting conceptualisation of the lived experience of personality disorders, illustrated in Fig. 1, adds to the limited existing literature and was characterised by:

- continuous tension within the self between experiences of an internal and an external world;
- complexity of ambiguous, interrelating and conflicting feelings, thoughts and actions as the individual tries to live in and cope with both worlds;
- feeling alienated by a hostile outside world and needing to isolate oneself within the internal world in order to feel safe;
- experience of receiving a diagnosis of personality disorders that could reinforce a sense of not fitting in with the outside world.

There are resonances in the limited experiential literature. Miller [35], in a Grounded Theory analysis of repeated, in-depth interviews with ten people diagnosed with Borderline Personality Disorder over a one year period developed themes of estrangement, inadequacy and despair from the interviews, with minimising self-disclosure and dissociation as coping strategies reported by participants. Castillo [11] trained a team of researchers with lived experience of personality disorders to conduct semi-structured interviews with 50 people with diagnoses of a range of personality disorders. Day to day experiences were characterised by interviewees in terms of rejection, isolation, self-blame, low self-esteem and unworthiness, while some described a connection between learning about their diagnosis and a sense of exclusion and hopelessness [11].

### Understandings of recovery and the lived experience of personality disorders

Through capturing this discourse of lived experience we were able to explore understandings of recovery that were relevant and meaningful to our interviewees (Fig. 2). Interviewees talked about recovery in terms of living in the outside world without having to isolate themselves continually in order to feel safe. That very specific understanding of recovery can be articulated as an expression of:

- increased awareness and acceptance of self, in part supported by better understanding that can come with diagnosis and treatment;
- reduction of conflict in the experience of internal and external worlds;
- internal and external worlds becoming reconciled (or at least the attempt to reconcile them);
- individuals thinking, feeling and acting differently in ways that enabled reconciliation between internal and external worlds to begin;
- recovery or, for some people, discovery of a sense of self that could begin to safely coexist in both internal and external worlds.

Aspects of this understanding of recovery – developing self-acceptance, gaining control over emotions and improving relationships – were also evident in the work of Katsakou and colleagues [20]. However the focus of that study was somewhat different to the analysis we report here; the Grounded Theory approach adopted by Katsakou and colleagues [20] shaped and refined emerging analysis to consider recovery in the context of symptoms and treatments associated with the specific diagnosis of Borderline Personality Disorder. In contrast Castillo, Ramon and Morant [21], as we did, considered recovery more specifically in the context of the lived experience of their participants and noted the importance of building safe and trusting relationships, alongside a sense of belonging and community, before more aspirational work can begin.

In our introduction we cite a conceptualisation of recovery borrowed from ‘mainstream’ mental health services that incorporated processes of empowerment, rebuilding positive identities, connectedness, hope, and finding meaning and purpose in life [3]. Equivalences of many of those processes are implicit in the understanding of recovery and personality disorders we present above. However there is inherent tension between our specific understanding of recovery and the particular value that mainstream understandings of mental health recovery place on social inclusion as facilitating a holistic sense of ‘living well’ with mental illness [36] and of engaging with society in order to move beyond the limitations of experiences defined by mental ill health [37]. What this mainstream understanding of social inclusion does not address is the experience of internal and external worlds, articulated by our interviewees, as in some way like poles of a magnet, with the potential to repel as well as to attract. Increased social interaction, building new relationships and engaging with wider society all invoked a sense of threat that could result in withdrawing from that interaction. Our interviewees seemed acutely aware that the steps they needed to take to move forward in their recovery also exposed them to potential harm to the self. Castillo, Ramon and Morant [21] acknowledge that their study might have excluded people who felt alienated by the sense of community that was central to the service from which their study participants were recruited.

Interviewees also told us how a vulnerable sense of self could be strengthened by positive experiences of interaction, and mainstream understandings of recovery report that positive social contact can be a source of validation of the individual’s growth and empowerment [38]. Yet for our interviewees this was very much uncharted territory; they seemed to be describing to us the *discovery*, rather than *recovery* of a sense of self that would enable them to live in both internal and external worlds. Some of Katsakou and colleagues’ [20] interviewees also questioned the appropriateness of the term *recovery*; they had experienced emotional difficulties for as long as they could remember, questioning implications in the term recovery of a pre-existing well state.

### Informing the development of personality disorders services

Our analysis cautions that existing conceptualisations of recovery borrowed from mainstream mental health services should not be applied, uncritically, to personality disorders services. Indeed comparisons between our study and those of Katsakou and colleagues [20], and Castillo, Ramon and Morant [21] suggest that understandings of recovery and personality disorders might be understood differently by different populations, reflecting the milieu of the service delivery environment from which participants were recruited.

Returning to the wider context of personalities disorders research, we note the still unresolved reconsideration of personality disorders as diagnostic labels [39, 40], alongside critiques of personality disorders diagnoses as morally driven codifications of behaviours disapproved of by society at large (e.g., [41]). In addition theories of gene-environmental interaction [42] as well as attempts to integrate neurobiological findings with theories of psychological development and psychodynamic understandings of personality disorders [43, 44] can all be viewed as indicative of a renaissance in efforts to better understand personality disorders.

Study of the discourse of lived experience has a role to play in this disparate yet creative process, and has been proposed a source of ‘resistance’ to the pathologising of the emotional distress experienced by women diagnosed with Borderline Personality Disorder [45]. More generally, within the field of medical anthropology it has been suggested that, through discourse about our health as we engage in medical treatment, we ‘resist’ the medicalisation of our bodies and coproduce our own local biologies [46]. The lived experience accounts we elicited are evidence of something similar. Our interviewees were articulating a lived discourse of personality disorders that could be described as resisting, or at least offering an alternative to mainstream, institutionalised thinking about recovery.

## Conclusions

Our analysis implies that any development of personality disorders services informed by concepts of recovery should be grounded in the discourse of lived experience of personality disorders. More specifically, as our analysis suggests, if key facilitators of recovery – positive personal relationships and wider social interaction – are also where an individual’s vulnerability lies, there is implicitly a role for personality disorders services in providing a safe space in which to develop those positive relationships. The wider personality disorders literature is indicative of the importance of the therapist-patient relationship in the success of both individual therapies [47] and psychosocial interventions [48]. An interpretive phenomenological study exploring the experiences of case management of 18 people diagnosed with Borderline Personality Disorder [49] identified ‘being treated like a person’ and a long term commitment to the individual as among the indicators of good case management. The role of the clinical practitioner in modelling positive relationships would seem to be an essential starting point in supporting recovery in personality disorders services. The need for staff working in personality disorders services to develop specialist skills – such as reflective practice and treatment alliance – to support people experiencing personality disorders has been acknowledged [7].

### Strengths and limitations of the research

The main limitation of our research was that interviewees were all recruited from the same specialist personality disorders service; understandings of recovery described here will necessarily have been shaped, to some extent, by interviewees’ common experiences of the peer support groups that comprised that service (as Castillo, Ramon and Morant [21] similarly acknowledged). Further qualitative research, using a more structured interview schedule derived from the conceptualisation of recovery we present here, would usefully recruit a larger sample from across a range of settings in order to establish the wider validity of our understanding.

Our collaborative, interpretive approach, described at the beginning of the paper, was a strength of the research. Throughout the analysis process the service user researcher on the team argued strongly that a lived experience perspective should guide and shape our analysis. We made explicit use of the service user researcher’s personal resonance with interviewees’ accounts to inform our descriptions of internal and external worlds experienced in conflict. The perspective brought to the research by the clinical researcher ensured that we foregrounded the importance of developing healthy relationships in supporting recovery. In reflecting on the research process we felt that it was our specific situated interpretations – informed by both personal, lived experience and experience of clinical practice – that enabled our analysis to move beyond a descriptive categorisation of participant accounts and offer a possible understanding of the processes of recover in the context of lived experience of personality disorders. Other research has similarly suggested that it is the reflection of the research team on ‘who we are’ as researchers that enables the coproduction of research knowledge [32]. Patterson and colleagues [24] describe a ‘dialogic collaborative process’ that stresses the importance of commitment on the part of the team to dialogue where there are tensions between perspectives. We suggest that it was our commitment to discursive practice as a research team that enabled us to capture the complex lived discourse of personality disorders described above. Through so doing we were able to produce an understanding of recovery that is both meaningful in the context of lived experience of personality disorders, and that might usefully inform the development of personality disorders services.

## Additional file

Additional file 1: **Developing an understanding of recovery for people with lived experience of personality disorders.** (DOC 49 kb)
