# Supplementary material for: Understanding recovery in the context of lived experience of personality disorders: a collaborative, qualitative research study
Source: BMC Psychiatry. 2015 Jul 31;15:183. doi: 10.1186/s12888-015-0572-0 (PMC4521354; doi:10.1186/s12888-015-0572-0)
Supplement: Additional file 1: — Developing an understanding of recovery for people with lived experience of personality disorders. (DOC 49 kb) [file 12888_2015_572_MOESM1_ESM.doc]

**Developing an understanding of recovery for people with lived experience of personality disorders**

**INTERVIEW SCHEDULE**

1. **First of all, could you tell me what the word ‘recovery’ means for you personally?**

Prompts:

- Recovery can mean different things to different people. We are interested in what it means to you.
- How do you know if you or someone else is recovered or recovering?

*Note to Interviewer:*

*If concept has no meaning to participant at the moment*:

- Recovery can be a broad concept covering many different areas. Which areas do you think are relevant?
- What might it mean to you in the future?
- How might it apply to you?

1. **What is your understanding of why you are being treated in this service?**

Prompts:

- Please can you tell me in your own words what mental health problems or other difficulties you think you have?

*Note to Interviewer:*

*If the participant is finding this difficult to answer:*

- Why do you think you are here?

1. **In relation to the (*use participant’s own words for describing mental health problems, or say “issues”*) you have mentioned, how much progress in recovery do you feel you have made?**

**What changes have there been so far?**

**How have those changes come about?**

**What else do you feel you have left to do?**

**How do the changes you’ve been telling us about compare with your expectations of recovery?**

1. **I would now like to ask you how you go about assessing where you are in your recovery**

**Firstly, what do you see as the signs of your recovery?**

**If you have a bad day / patch, how does that affect your feelings about recovery?**

**How would you go about judging the success of your recovery?**

**How do the opinions of others relate to your assessment of your own recovery?**

1. **I’d now like to ask about the factors that have contributed to your recovery. By this we mean anything that has been important to your recovery, or that you think is related to your recovery**

**Firstly, what do you think are the factors that helped to start or trigger your recovery (if any)?**

**Secondly, what are the factors that help to support or sustain your recovery?**

**And are there any factors that have hindered or set back your recovery?**

**What factors in your recovery have been most important?**

**6. I’d now like to ask about aspects of the treatment you have received in connection with the issues you talked about earlier. By ‘treatment’ we mean the different types of help you’ve had, both now and in the past.**

*Note to Interviewer:*

*If treatment has been covered in Q5, ask if there are any other aspects of treatment that have helped recovery.*

**What aspects of treatment, if any, have helped your recovery?**

*Note to Interviewer:*

*If no response to the above:*

- Can you think of any aspect of your treatment that has been, or is, helpful to you in your recovery?

**Were there any treatments outside *the service you are in* that you feel were particularly helpful?**

**Overall, which were the necessary parts of treatment in your opinion?**

**7. What aspects of the treatment you have received do you feel have had no impact upon your recovery?**

*Note to Interviewer:*

*Redefine treatment if necessary.*

Prompts:

- Which aspects of your treatment made no difference to your progress?
- Are there any types of treatment you could have managed without?
- Were there any types of treatment you felt were a waste of time?

**8. What aspects of the treatment you have received have prevented or set back your recovery, if any?**

Prompts:

- Were there any treatments you feel caused you harm?
- Were there any treatments you felt got in the way of your recovery?
- Were there any treatments you felt undid some of the progress that you had made?
- In retrospect if you could make some changes to the treatments you have received, what would they be?
